# Femtosecond laser-assisted cataract surgery after penetrating keratoplasty: a case report

**DOI:** 10.1186/s12886-017-0496-1

**Published:** 2017-06-24

**Authors:** Danmin Cao, Shiming Wang, Yong Wang

**Affiliations:** 1 Wuhan Aier Eye Hospital, Aier Eye Hospital Group, Wuhan, China; 2 Aier School of Ophthalmology, Central South University, Changsha, China; 3 Ningbo Aier Guangming Eye Hospital, Aier Eye Hospital Group, Ningbo, China

**Keywords:** Femtosecond, Cataract, Corneal, Transplantation

## Abstract

**Background:**

Cataract surgery after penetratingkeratoplasty (PKP) is often challenging due to changes in the integrity of the cornea caused by PKP. For example, corneal endothelial cell (CEC) loss and corneal edema commonly occur after traditional phacoemulsification cataract surgery in patients that previously had successful PKP. Recent studies have reported that femtosecond laser-assisted cataract surgery (FLACS) significantly reduces the need for ultrasound energy minimizing mechanical damage to the cornea and results in a reduction of CEC loss and corneal edema.

**Case presentation:**

We report a case in which FLACS was used in a patient with previous PKP.

**Conclusion:**

This case supports the suggestion that the use of the femtosecond laser improves the surgical outcome of cataract surgery after PKP. This improvement may be result of the precise incision, controlled capsulorhexis, and reduced lens fragmentation experienced with the femtosecond laser which helps to reduce potential complications of cataract surgery after PKP.

## Background

Previous studies report that 44–64% of patients develop cataracts within five years of PKP. Cataract surgery after PKP is often challenging due to changes in corneal integrity induced by PKP. CEC loss and corneal edema often occur after traditional phacoemulsification cataract surgery in patients that had previous successful PKP [1]. The mean annual rate of endothelial cell loss from 10 to 15 years after surgery was 0.2 +/− 5.7% [2].

Corneal distortion, irrigation solution turbulence, instrument-related mechanical trauma, nuclear fragments, IOL contact, and free oxygen radicals can cause corneal damage during cataract surgery. Several preoperative and intraoperative parameters (high nucleus grade, advanced age, long phaco time, high ultrasound energy, short axial length, and surgical skill) are associated with an increased risk of endothelial cell damage after phacoemulsification [3]. Recent studies had shown that the use of FLACS significantly reduces the need for ultrasound energy minimizing mechanical damage to the cornea and results in a reduction of CEC loss and corneal edema [4].

## Case presentation

**Fig. 1:**
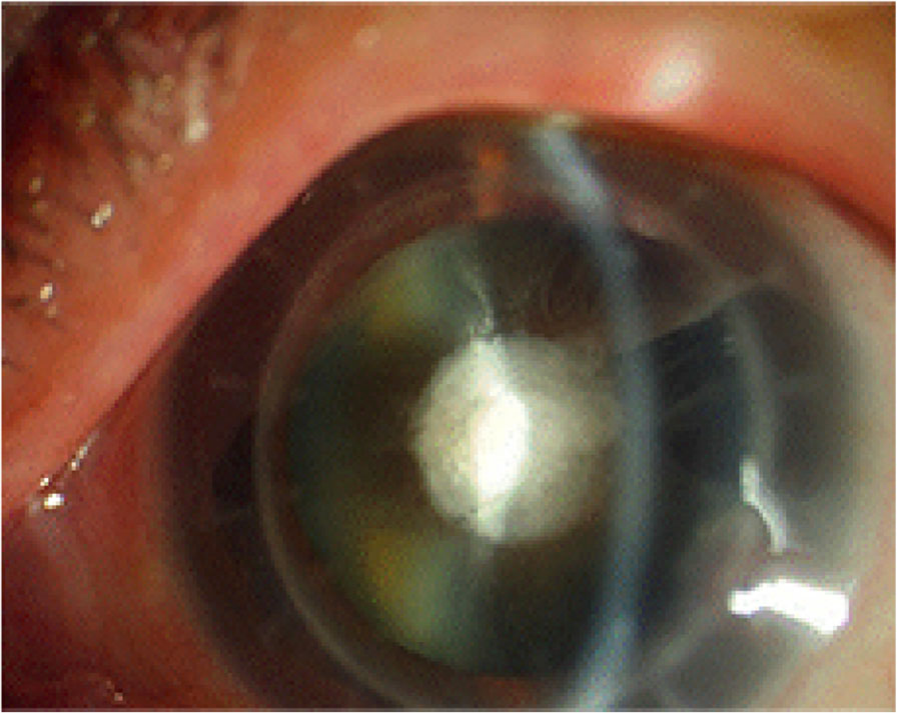
A preoperative slitlamp image showing a corneal graft transparency and a hard cataract with a 3 mm centered *white* anterior lens capsule calcification

**Fig. 2:**
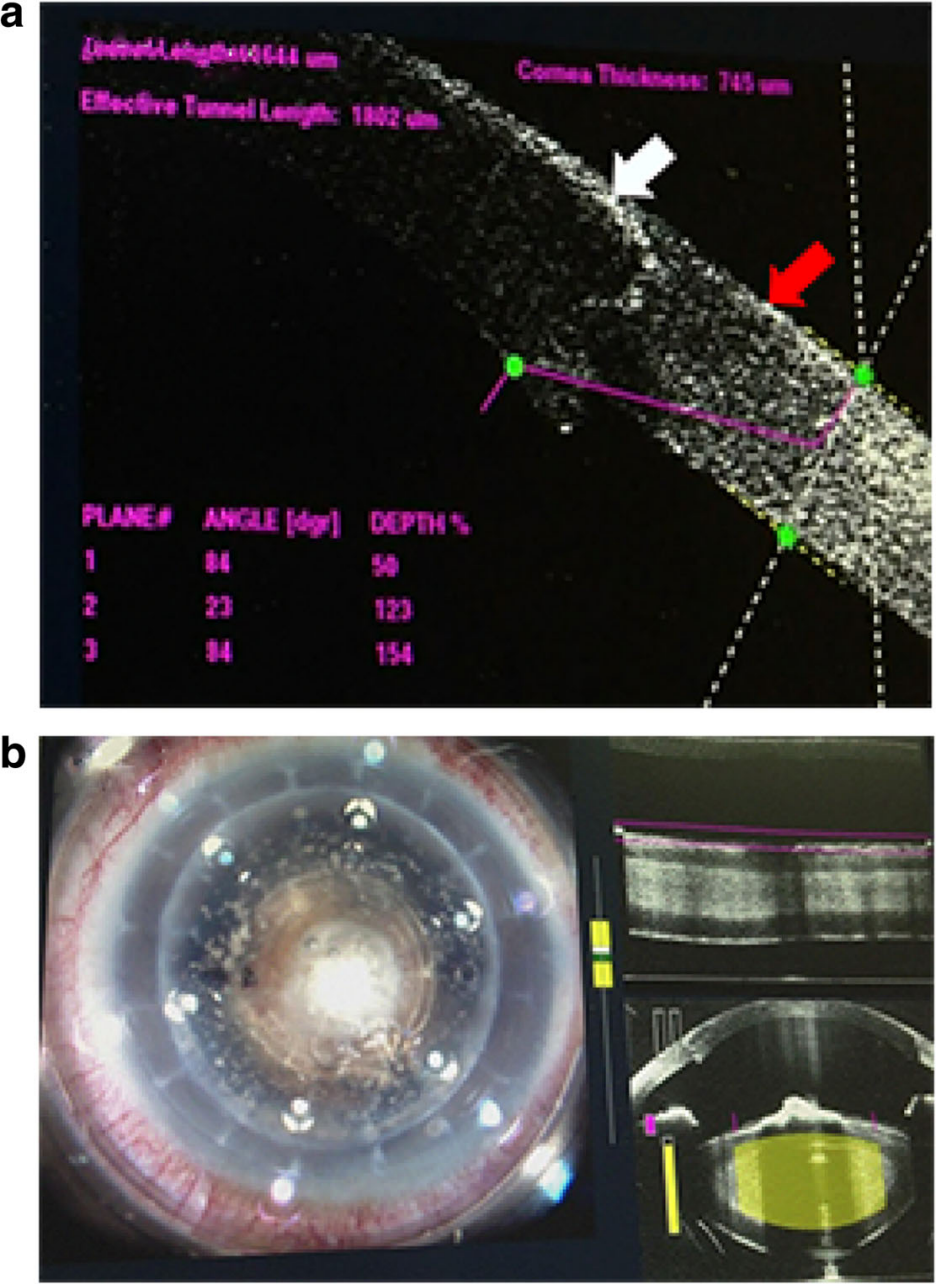
**a** AS-OCT image depicts the architecture of clear corneal incision. The *white arrow* indicates the corneal graft. The *red arrow* indicates corneal bed. **b** A 5.0 mm capsulotomy diameter was selected. The AS-OCT indicated that the anterior lens capsule calcification of the central areas had a bulge

A male patient of 61-years that had 7.0 mm diameter PKP performed in his left eye two years ago. His best corrected visual acuity (BCVA) at distance in the left was light perception. Slit lamp examination showed a corneal graft transparency and a hard cataract with a 3 mm centered white anterior lens capsule calcification (Fig. 1). Due to the hardness of cataract in this case, the IOL-Master could not assess the axial length (AL) and an A-Scan (TOMEY, AL2000, Japan) was used to assess AL. Keratometry was assessed using the auto-refractometer (Topcon, KR8800, Japan).IOL power was calculated using the SRK/T formulas, using the A-Scan software. The LenSx laser system (Alcon Laboratories, Inc.) was used to perform this surgery. Based on this examination, a 5.0 mm capsulotomy diameter was selected. Anterior segment optical coherence tomography (AS-OCT) showed a bulge in the anterior lens capsule calcification of the central area. An AS-OCT-guided 2.2 mm corneal incision was created (Fig. 2a). Cylinder and chop pattern was used for lens fragmentation (Fig. 2b). After completion of the laser procedure, the patient was moved to the operating room. The surgery was completed with a standard phacoemulsification procedure using the Infiniti Vision System. The Cumulative Dissipated Energy (CDE) was used to assess phacoemulsification time and phacoemulsification power. The CDE was 8.69, the total surgical time was 16 mins., (from femtosecond laser to watertight), and the volume of irrigating solution used was 92 ml. A 23D Acrysof SN60WF intraocular lens(IOL) (Alcon Laboratories, Inc.) was implanted.

**Fig. 3:**
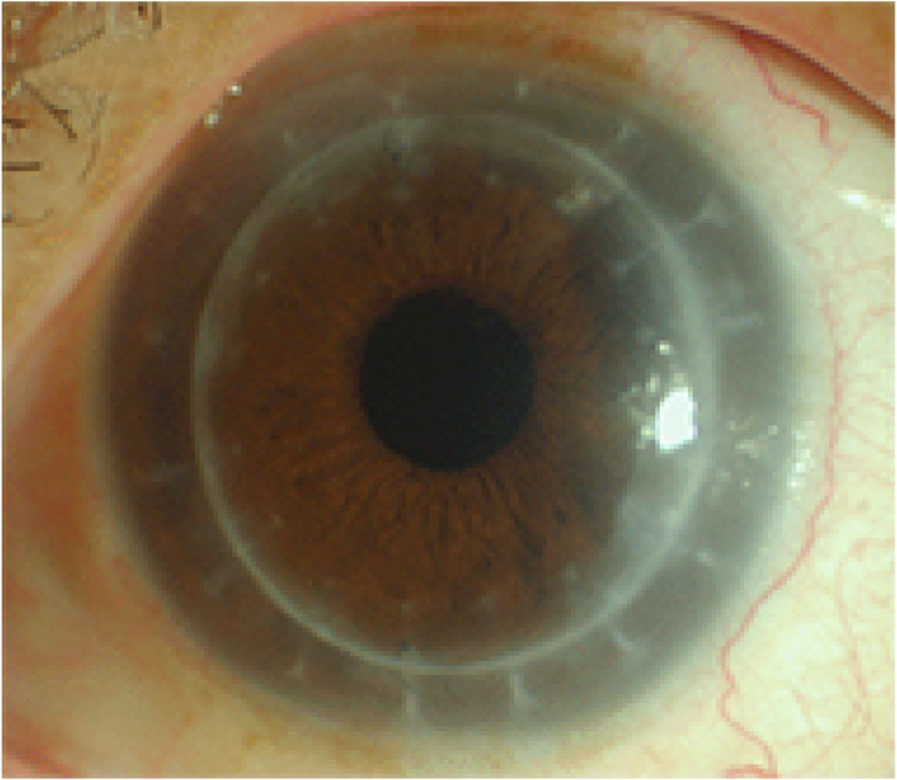
A postoperative slitlamp image showing a transparent cornea and a well-centered IOL

On postoperative day 1, the uncorrected visual acuity (UCVA) was 20/40 with a transparent cornea and a well-centered IOL with a 360-degree capsule overlap (Fig. 3). The postoperative UCVA improved to 20/25 after one month. Subjective refraction was stable at 0.75 D sphere and 2.00 D cylinder at the three month follow up. The specular microscope (Topcon, SP2000P,Japan) was used to measure CEC numbers. Preoperative CEC numbers were 1947 cells/mm^2^, 1792 cells/mm^2^ immediately after surgery, 1628 cells/mm^2^ at one month and 1517 cells/mm^2^ at three months.

## Discussion and conclusions

The three critical steps for successful cataract surgery after PKP areobtaining a central continuous capsular capsulotomy, minimizing ultrasound energy and ensuring a closed incision. Manual capsulorhexis is a significant challenge, such as in this case, when an intumescent cataract is present with a 3 mm centered white anterior capsule. Many studies report a decreased rate of anterior capsule tears in FLACS compared to manual phacoemulsification cataract surgery [5–7]. Additionally, the creation of a precise, safe, and reproducible capsulotomy is a prerequisite for successful cataract surgery and IOL implantation. Compared to manual capsulorhexis [8], the femtosecond laser has been shown to create a particularly well-shaped and reproducible capsulotomy geometry and circularity [9]. In this case, intraoperative AS-OCT of LenSx was able to image through white opacities of the lens anterior capsule calcifications in advanced cataracts [10]. The femtosecond laser capsulotomy diameter is highly controlled. In this case, the diameter was set to 5 mm, larger than the 3 mm central area of the anterior lens capsule calcification. Capsulotomies performed by the femtosecond laser ensures the safety of intraoperative nucleus chopping and provides accurate location of the IOL [11]. Dick, et al. compared the surgical outcome of patients who underwent the standard manual phacoemulsification versus FLACS. They report that the FLACS group had significantly less capsular bag shrinkage than the standard group at one, two, and three months, with a mean difference of 0.33 ± 0.25 mm at three months [12]. Early stabilization of the capsular bag diameter leads to more predictable effective lens position, IOL power calculations, and refractive outcomes.

Normal function of corneal endothelial cells is essential for maintaining corneal transparency [13, 14]. Cataract surgery after corneal transplant must minimize endothelial cell damage as the transplant has fewer endothelial cells compared to normal corneas. In our case, the preoperative of CEC numbers were 1947 cells/mm^2^ and a decrease in endothelial cell numbers occurred after phacoemulsification [15]. Several studies [16–18] report a direct relationship between endothelial cell loss and ultrasound power and time. Endothelial cell loss related to ultrasound use is markedly higher in cornea graft than in normal corneas [19]. Furthermore, the cataract nucleus hardness in this case was a grade IV .To perform this procedure with traditional phacoemulsification would require more energy and significantly decrease postoperative CEC numbers. Using extra capsular cataract extraction (ECCE) causes less endothelial cell loss compared to phacoemulsification [17], but often induces an astigmatism which leads to poor vision. Femtosecond laser uses ultra short pulses of near infrared light to disrupt tissue with micron precision, this minimizing tissue damage [20]. Lens fragmentation, induced by the femtosecond laser, significantly reduces endothelial cell damage by minimizing the amount of potentially injurious ultrasound energy required to emulsify the lens. Furthermore, laser energy is focused on the capsular bag and limits exposure to the endothelium [21]. Here, we report that our patient had postoperative CEC numbers of 1517 cells/mm^2^ at the three-month follow up.

The thickness of the corneal graft and corneal bed are often not the same which leading to poor incision architecture during traditional phacoemulsification. Femtosecond laser improves incision architecture by increasing the precision and reducing mechanical and thermal stress at the incision site [22]. In our case, the corneal incision was made by femtosecond laser and guided by AS-OCT.

In conclusion, the use of femtosecond laser allows precise incisions, controlled capsulorhexis and reduces the amount of ultrasound energy required for lens removal. This technique reduces potential complications in cataract surgery after PKP and improves visual recovery and refractive results.

## Abbreviations

AC-OCT: Anterior segment optical coherence tomography
BCVA: best corrected visual acuity
CEC: corneal endothelial cell
FLACS: femtosecond laser-assisted cataract surgery
IOL: intraocular lens
PKP: penetratingkeratoplasty
UCVA: uncorrected visual acuity
